# Quality of transition to end-of-life care for cancer patients in the intensive care unit

**DOI:** 10.1186/s13613-015-0059-7

**Published:** 2015-07-25

**Authors:** Sophie J Miller, Nishita Desai, Natalie Pattison, Joanne M Droney, Angela King, Paul Farquhar-Smith, Pascale C Gruber

**Affiliations:** Palliative Care Department, Royal Marsden Hospital, The Royal Marsden NHS Foundation Trust, London, UK; Critical Care Unit, Intensive Care Unit, Royal Marsden Hospital, The Royal Marsden NHS Foundation Trust, London, SW3 6JJ UK; Medical Oncology, Royal Marsden Hospital, The Royal Marsden NHS Foundation Trust, London, UK

**Keywords:** Palliative, Intensive care, Communication, Do not resuscitate

## Abstract

**Background:**

There have been few studies that have evaluated the quality of end-of-life care (EOLC) for cancer patients in the ICU. The aim of this study was to explore the quality of transition to EOLC for cancer patients in ICU.

**Methods:**

The study was undertaken on medical patients admitted to a specialist cancer hospital ICU over 6 months. Quantitative and qualitative methods were used to explore quality of transition to EOLC using documentary evidence. Clinical parameters on ICU admission were reviewed to determine if they could be used to identify patients who were likely to transition to EOLC during their ICU stay.

**Results:**

Of 85 patients, 44.7% transitioned to EOLC during their ICU stay. Qualitative and quantitative analysis of the patients’ records demonstrated that there was collaborative decision-making between teams, patients and families during transition to EOLC. However, 51.4 and 40.5% of patients were too unwell to discuss transition to EOLC and DNACPR respectively. In the EOLC cohort, 76.3% died in ICU, but preferred place of death known in only 10%. Age, APACHE II score, and organ support, but not cancer diagnosis, were identified as associated with transition to EOLC (*p* = 0.017, *p* < 0.0001 and *p* = 0.001).

**Conclusions:**

Advanced EOLC planning in patients with progressive disease prior to acute deterioration is warranted to enable patients’ wishes to be fulfilled and ceiling of treatments agreed. Better documentation and development of validated tools to measure the quality EOLC transition on the ICU are needed.

## Background

Outcomes for critically ill cancer patients have improved over the last decade, and intensivists are increasingly willing to initiate a ‘trial of intensive care unit (ICU) therapy’ in patients with advanced cancer [1, 2]. The aim of a trial of intensive care therapy is to support patients’ recovery, treat reversible causes of illness and help patients survive immediate threats to their lives. It is estimated that 18–30% of cancer patients use of intensive care services [3, 4]. Advances in cancer diagnosis, treatment and general ICU care have led to better outcomes in patients with cancer admitted to the ICU [1, 2]. However, morbidity and mortality still remains high, with cancer patients admitted to ICU having a mortality of 27–43% [5–7]. Recent studies have shown similar ICU mortality rates for patients with solid tumours and haematological malignancies [2, 8, 9].

The transition from intensive care with the goal to save or prolong life to end-of-life care (EOLC) can often be a difficult decision to make for clinicians, patients and families [10]. Clinical parameters to help clinicians identify patients who are likely to transition to EOLC during their ICU stay are often not easily defined [9, 11]. The ability to identify clinical parameters early in their ICU admission that could help predict likelihood of transition to EOLC could improve the quality of care for cancer patients.

Previous studies have demonstrated that care pathways and communication during transition to EOLC for critically ill patients and their families in the ICU setting is not always optimal [12–15]. Problems reported have been poor communication; discordance about treatment plans and goals; high prevalence of pain and distressing symptoms [16–19]. Healthcare providers also highlight a lack of knowledge and training in EOLC [20]. Furthermore, it is unclear which, if any, palliative measures are effective, or increase satisfaction, in the ICU [21]. Clarke et al. and a subsequent consensus statement from the American College of Critical Care Medicine recommended seven quality indicators (Table 1) for improving the EOLC of ICU patients during the dying process [15, 22].

**Table 1:** American College of Critical Care Medicine key recommendations for improving the care of ICU patients during the dying process: adapted from Clarke et al. [22] and Mularski et al. [23]

1. Patient- and family-centred decision-making	
Involve patient and family in decision-making/discussions as appropriate to the individual; initiate advanced care planning	
2. Communication with team/patient/family	
Meet with the multidisciplinary team to plan care; meet regularly with patient and family to review situation and answer questions; sensitive communication	
3. Spiritual support	
Regularly assess and document spiritual needs; document offer of spiritual support	
4. Emotional and practical support	
Open visitation for family; provide support and written logistical information (e.g. accommodation); financial and bereavement advice	
5. Symptom management and comfort care	
Assess symptoms before and after interventions; follow best clinical practice using pharmacologic and non-pharmacologic means for best symptom management	
6. Continuity of care	
Maximise continuity of care for patients; introduce new clinicians	
7. Emotional and organisational support for intensive care unit clinicians	
Support and educate staff on the ICU who are caring for dying patients	

The primary aim of this study was to explore the quality of transition to EOLC for medical patients with cancer who died, or transitioned to end-of-life on the ICU. Medical patients only were sampled, as the predicted and actual mortality for surgical patients in the unit was historically very low, and few transitioned to end-of-life [2]. The literature attests to the high mortality in medical ICU cancer patients, and low surgical mortality [24, 25]. The secondary aim was to identify parameters on ICU admission that could help determine patients who were likely to transition to EOLC during their ICU stay.

## Methods

### Setting

This study was approved by the local Committee for Clinical Research. The study took place on the ICU in a 269-bedded specialist adult cancer hospital located across two sites in London. The hospital serves the local population and is also a tertiary referral centre for patients with cancer from across the United Kingdom and abroad. The 16-bedded mixed medical and surgical ICU admits approximately 1,400 patients per year and has capacity for both level 2 (single organ support, or extensively post-operative care) and 3 (two or more organ support, or advanced respiratory support) care [26]. All referrals and admissions to ICU are discussed with the ICU consultant. No formal EOLC protocol was in place in the ICU during the study period following the abolition of the Liverpool Care Pathway [27]. However, several of the recommendations for improving communication at the end-of-life by the American College of Critical Care Medicine had been adopted in the unit [15]. These included formal communication skills training of ICU and Palliative Care Consultants, interdisciplinary team rounds, palliative care team input at weekly ICU multidisciplinary team meetings and regular family meetings.

All medical patients with cancer admitted to the ICU over a 6-month period from 01.03.2013 to 01.08.2013 were included in this study. Patients <16 years, without a cancer diagnosis, or who had undergone elective or emergency surgery were excluded as the focus on this study was primarily on medical cancer patients. For those patients who had more than one admission during the study period, only the patient’s last admission was included in the analysis. Sepsis was defined according to the Surviving Sepsis Guidelines [28].

### Study design

A retrospective review of patient records was carried out to explore patients’ demographics and co-morbidities; cancer type and stage; reason for hospital and ICU admission; documented communication between teams, patients and relatives (detailed from care records, team meetings and daily ward round annotations in the electronic patient records); documented palliative care team involvement; documented evidence of resuscitation and ceiling of care decisions; documented quality of EOLC provision, and ICU mortality.

To determine the care process and quality of EOLC transitions on the ICU, quality indicators recommended by American College of Critical Care Medicine were used in the qualitative documentary analysis (Table 1) [15]. The quantitative variables were categorised into process and outcome domains. The first five points (Domains 1–5) were used in the analysis on processes of EOL transition and provision of EOLC in our ICU. Communication not only included documented presence of patient, family and team members, but also in some cases, a qualitative analysis of the documented discussion. At the time of this study, standardised symptom assessment tools were not routinely used or integrated into the electronic documentation. Symptom control (Domain 5) was defined, for simplicity, as documented evidence of pharmacological and non-pharmacological control of symptoms such as pain, nausea, vomiting, diarrhoea, and anxiety. Assessments were made prior and after intervention to determine if patients’ had achieved adequate symptom control. Domains 6 and 7 were excluded as they relate to organisational processes that could not be determined through patient records review. Palliative or non-curative treatment intent was defined as patients with no further active treatment options available, but those that could be on phase 1 trials or be receiving treatment to control or maintain disease stability but without curative intent. In contrast, curative intent was defined as patients who were receiving active treatment for their cancer with an intention of cure [29].

Patient records were examined using the Electronic Patient Record (EPR), IntelliView Clinical Information Portfolio (ICIP), chaplaincy database, and written notes. All data was double checked for accuracy by two investigators. All data were entered onto an excel database and collated into 46 variables under the aforementioned process and outcome categories. These variables were then categorised and tabulated into the five domains (Domains 1–5). Data were pseudo-anonymised, and dealt with Good Clinical Practice guidelines and United Kingdom Data Protection Act.

### Identification of cohorts: ‘EOLC’ vs ‘full active ICU care’

**Fig. 1:**
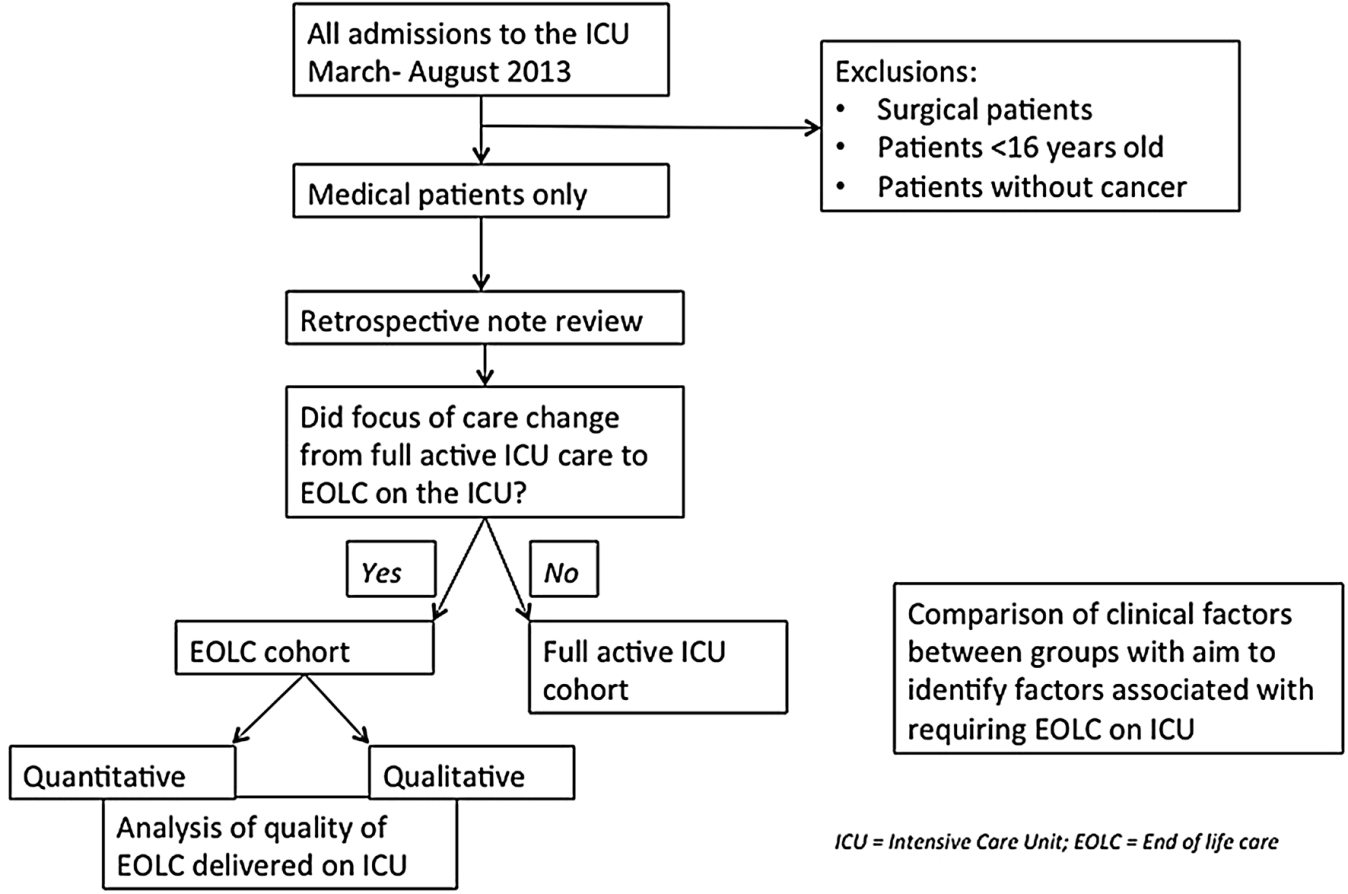
Flowchart of protocol for patient selection and analysis.

Following data collection, patients were divided into two cohorts: patients who received full active management with organ support throughout their ICU stay and whose main focus of care remained prolongation of survival with curative intent (‘full active ICU care’ cohort); and those patients who had transitioned from full active management to EOLC during their ICU stay or died on the ICU (‘EOLC’ cohort) (Fig. 1). The transition to EOLC was ascertained on an individual patient basis and decisions were made when the teams (parent medical team, ICU team and sometimes the palliative care team) jointly concluded that continuation of life-prolonging organ support would be futile and not in the patients’ best interests. Families (and patients, if they had capacity) were involved in these discussions. It is recognised however, that some of EOLC cohort may have received life-prolonging organ support during the early part of their ICU stay before transitioning to EOL care. Equally, symptom control was given in the full active care cohort where needed.

### Data analysis

Quantitative data were analysed using SPSS statistics software (version 20, Chicago, IL, USA). Comparison was made between the two cohorts using standard descriptive statistics. For categorical variables such as organ support required in ICU and performance status, Fisher’s exact test and Chi squared tests were performed to compare data between the two groups. Continuous data was analysed using the Mann–Whitney *U* (for non-parametric data including age) and student *t* test for parametric data. A statistical significance *p* < 0.05 was used when comparing the two cohorts.

Multiple logistic regression was used to investigate the joint effect of clinical variables on the whether or not patients transitioned to the EOLC cohort or not. Factors with *p* < 0.1 on univariate analysis were included in the multivariate modelling. Variables with *p* > 0.1 were excluded in order to reduce the number of predictor variables. Variable selection was carried out using a stepwise method, with *p* < 0.05 and *p* > 0.1 as criteria for entry and removal, respectively. Only factors with *p* < 0.05 were retained in the final model.

Data from the EOLC cohort were further analysed using descriptive statistics to explore the quality of EOLC being delivered on the ICU.

Text data from all medical records (ICIP and hospital EPR) was analysed using qualitative thematic analysis. Undertaking textual analysis enriched the quantitative data yielded [30, 31]. A mixed analysis allowed examination and exploration of unusual quantitative findings [32]. Thematic analysis provided a structure for identifying patterns in data and involved steps of data codification; testing and connecting codes, searching and establishing themes; reviewing and corroborating themes.

## Results

There were 3,203 hospital in-patient admissions during the study period, and of these 669 were admitted it the ICU (20.9%). Of the ICU admissions, 85 were medical patients (12.2%), all of whom were included in the analysis.

### Quantitative analysis comparing ‘Full active ICU care’ with ‘EOLC’ cohorts

Of the 85 patients analysed, 38 patients (44.7%) met the criteria for ‘EOLC’ and 47 patients (55.3%) remained for ‘full active ICU care’. Patient demographics are outlined in Table 2. Median age and APACHE II score was higher in the EOLC group compared to the full active ICU care group (66.5 vs. 59 years, *p* = 0.017) and (21 vs. 16, *p* < 0.0001), respectively. The EOLC cohort required a higher level of organ support (intubation 44.7 vs. 12.8%, *p* = 0.001; vasopressors 55.3 vs. 27.7%, *p* = 0.014 and RRT 28.9 vs. 8.5%, *p* = 0.021). There was no statistical difference in cancer type, disease stage or co-morbidities. On multivariate analysis, APACHE II score, the need for intubation and non-invasive ventilation remained significantly associated with a poor prognosis (Table 3).

**Table 2:** Comparison of characteristics between the ‘Full active ICU care’ cohort and the ‘EOLC’ cohort

Characteristic	Full active ICU care *n* = 47	EOLC *n* = 38	*P* value
Age, median (range) in years	59 (19–88)	66.5 (28–81)	*0.017*
Gender, *n* (%)	M 21 (44.7%)	M 19 (50.0%)	0.62
	F 26 (55.3%)	F 19 (50.0%)	
APACHE II score median (range)	16 (5–31)	21 (10–47)	*<0.0001*
Type of cancer, *n* (%)			
Solid tumour	35 (74.5%)	24 (63.2%)	0.26
Haematological tumour	12 (25.5%)	14 (36.8%)	0.26
Stage of disease, *n* (%)*			
No known spread	7 (14.9%)	6 (15.8%)	0.92
Local spread (spread to neighbouring tissues)	7 (14.9%)	2 (5.3%)	0.14
Nodal spread (spread to lymph nodes)	7 (14.9%)	5 (13.2%)	0.82
Distant metastases	23 (48.9%)	21 (55.3%)	0.47
Cancer treatment, *n* (%)			
Chemotherapy	32 (68%)	29 (76.3%)	0.4
Radiotherapy	11 (23.4%)	4 (10.5)	0.16
Palliative treatment (definitions in text)	19 (43.1%)	17 (44.7%)	0.69
Curative treatment (definitions in text)	22 (46.8%)	16 (42.1%)	0.66
No treatment	6 (12.8%)	3 (7.9%)	0.51
Reason for ICU admission, *n* (%)**			
Respiratory failure	19 (40.4%)	17 (44.7%)	0.69
Renal failure	10 (21.3%)	9 (23.7%)	0.79
Cardiac	7 (14.9%)	3 (7.9%)	0.5
Sepsis	11 (23.4%)	12 (31.6%)	0.4
Neurology	2 (4.3%)	1 (2.6%)	0.69
Other	8 (17.0%)	7 (18.4%)	0.73
Time of referral to ICU, *n* (%)			
Out of hours	22 (46.8%)	16 (42.1%)	0.82
Weekend	11 (23.4%)	6 (15.8%)	0.55
Organ support required during ICU stay, *n* (%)			
Invasive ventilation	6 (12.8%)	17 (44.7%)	*0.001*
Vasopressors	13 (27.7%)	21 (55.3%)	*0.014*
Renal replacement therapy (RRT)	4 (8.5%)	11 (28.9%)	*0.021*
Non-invasive ventilation (NIV)	9 (19.1%)	12 (31.6%)	0.19
Baseline performance status documented, *n* (%)	18 (38.3%)	16 (45.7%)	0.72
Need for symptom control in ICU, *n* (%)	27 (57.0%)	27 (71.0%)	0.19
Seen by palliative care in ICU, *n* (%)	7 (19.1%)	20 (52.6%)	*0.001*
Cancer prognosis documented prior to ICU, *n* (%)	10 (21.2%)	4 (10.5%)	0.15

Statistically significant variables are in italics

* 3 patients in ‘full active ICU care’ cohort and 4 patients in ‘EOLC’ cohort had leukaemia and therefore were excluded from this analysis.

** Some patients had more than one reason for admission hence total numbers greater than 100% per cohort.

**Table 3:** Multivariate analysis of factors associated with transition to EOLC

Multivariate logistic regression model of factors associated with patients transitioning to EOLC in ICU		
Variable	*P* value	OR (95% CI)
APACHE II score	0.003	1.144 (1.046–1.251)
Invasive ventilation	0.012	0.206 (0.06–0.703)
Non-invasive ventilation	0.027	0.269 (0.084–0.863)
Constant	0.545	0.500

Overall ICU mortality was 34% for all medical patients admitted to the ICU and 3.7% (118/3,203 patients) for all hospital in-patients during the study period.

### Quantitative analysis of the quality of transition to EOL

Of the 38 patients who were included in the EOLC cohort, only one patient was admitted for symptom control alone. All other patients were initially admitted for full active ICU management.

Most patients in the EOLC cohort (37/38 patients; 97.4%) had clear documented evidence of transition from full active care to EOLC.

### ICU and in-hospital mortality

Most patients (29/38 patients; 76.3%) in the EOLC cohort died on the ICU. Of the remaining 9 patients, 7 died on the ward; one died in hospice and one was transferred to their home country whilst remaining intubated. In-hospital mortality for the EOLC cohort was (36/38 patients; 94.7%).

### Resuscitation decisions

On admission to ICU most patients in the EOLC cohort (34/38 patients; 89.5%) were for full resuscitation. In the EOLC cohort ‘do not attempt cardiopulmonary resuscitation’ (DNACPR) orders were completed for 47% patients within 48 h of ICU admission. Most patients (97.4%) in the EOLC cohort had documented evidence of DNACPR at some point during their ICU stay. Sixty-nine percent of patients died within 3 days of DNACPR decision. The time from DNACPR decision to death was a median of 1.5 days (range 0–93 days). In contrast, amongst the ‘full active ICU care’ cohort, just 2/45 patients (4.4%), had a DNACPR order documented during their ICU stay.

### Documented quality markers for transition to EOLC

The quality of EOLC delivered during transition to end-of-life care was assessed using the domains adapted from the American College of Critical Care Medicine Consensus statement, is shown in Table 4 [15, 22, 23].

**Table 4:** Quality markers for EOLC on the ICU

Quality markers for end-of-life care on the intensive care unit	% (*N*/total *N*)
Symptom management and comfort care	
Documented evidence of need for symptom control as evidenced by the documented evidence of symptoms such as pain, shortness of breath, anxiety, nausea, vomiting, constipation	71 (27/38)
Documented evidence of successful symptom control (*N* = 27)*	79 (21/27)
Documented evidence that the patient was reviewed by the hospital specialist palliative care team	53(20/38)
Reason for referral to hospital specialist palliative care team (*N* = 20)**	
Symptom control	80 (16/20)
EOLC	80 (16/20)
Psychosocial support	25 (5/20)
Communication with team, patient and family	
Documented evidence that a professional decision had been made that life and organ support was no longer feasible or appropriate and that these therapies were going to be withdrawn or withheld and that the likelihood of death was high	44 (37)
Is there documented evidence that this decision had been discussed with the patient, relative and oncology team	
Discussed with patient	43 (16/37)
Not possible to discuss with patient being too unwell	51 (19/37)
No record of whether or not discussed with patient	5 (2/37)
Discussed with relative	97 (36/37)
Discussed with parent oncology team	92 (34/37)
Documented evidence that a professional decision had been made that the patient should not be for cardiopulmonary resuscitation in the event of a cardiopulmonary arrest (DNACPR order completed)	44 (37)
Documented evidence that this decision was:	
Discussed with patient	41 (15/37)
Not possible to discuss with patient as too unwell	41 (15/37)
No record of whether or not discussed with patient	19 (7/37)
Discussed with relative	89 (33/37)
Discussed with parent oncology team	73 (27/37)
Patient- and family-centred decision-making	
Documented evidence that the patient had an advance directive or an Advanced Decision to Refuse Treatment in place	0 (0/38)
Documented evidence about the patient’s wishes and preferences for their preferred place of death	11 (4/38)
Emotional and practical support	
Documented evidence that psychological support was offered to the patient	29 (11/38)
Documented evidence that psychological support was offered to relatives	21 (8/38)
Documented evidence that practical and welfare advice (e.g. about welfare benefits/accommodation) was offered to the patient/relatives	21 (8/38)
Spiritual support	
Documented evidence that a discussion took place with the patient or family regarding their spiritual needs or that chaplaincy support was offered	37 (14/38)

*DNACPR* do not attempt cardiopulmonary resuscitation.

* Symptom control by interventions delivered by any medical team, not just specialist palliative care team.

** Often referred for more than one reason.

### Qualitative thematic analysis

**Fig. 2:**
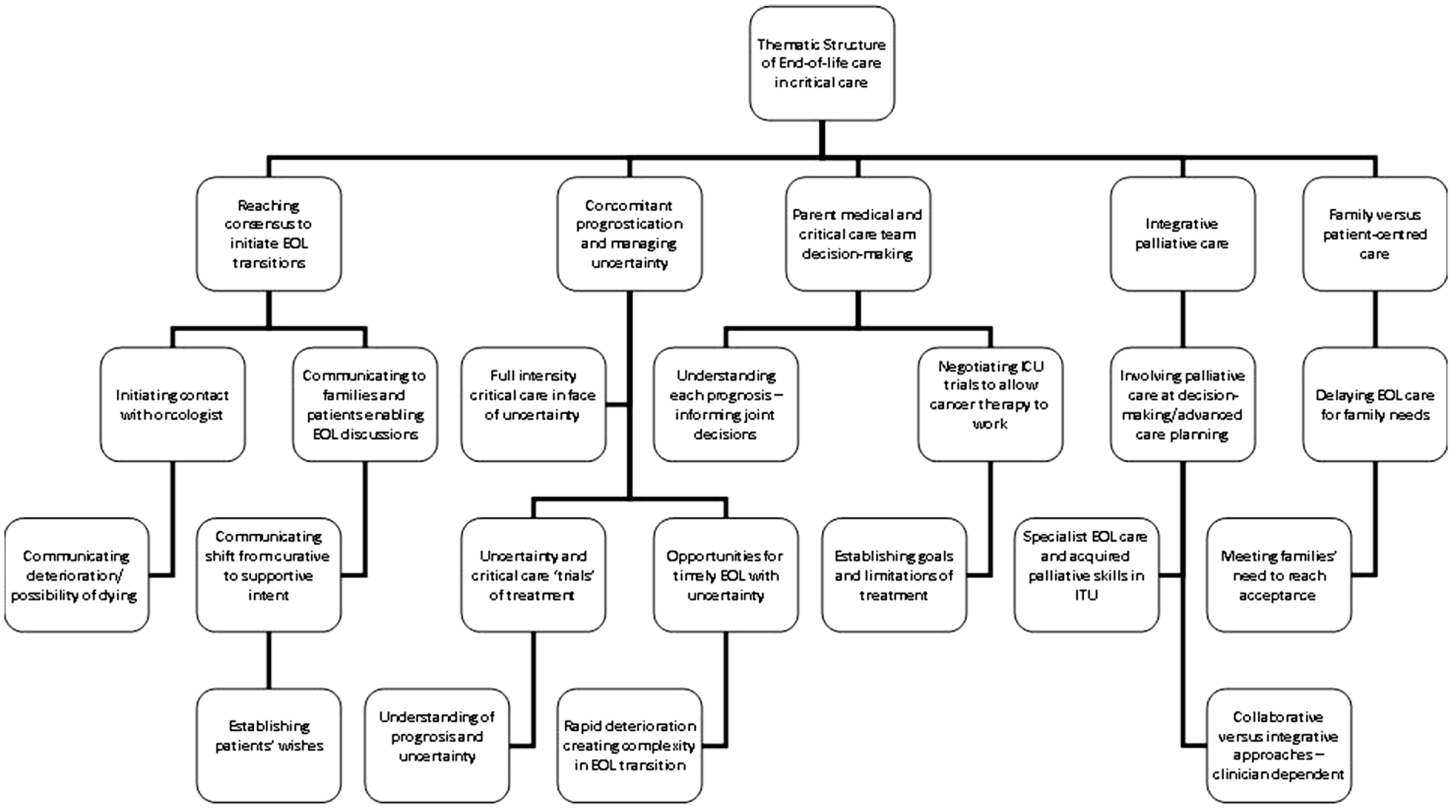
Thematic structure of end-of-life care in Critical care.

Data were analysed qualitatively for all patients under a priori framework analysis categories of prognostication, decision-making and EOLC based on the quantitative findings. These were modified according to the 19 sub-themes (Fig. 2) and resulted in five main themes (Table 5).

**Table 5:** Exploring the 5 main themes

*1. Achieving a consensus to initiate end of life transitions*	
1.1 ‘I have discussed his management with [oncologist] this morning. He agrees that it would not be appropriate to start [mechanical ventilation] [. . .] It is unlikely he will survive beyond the next few hours and even with full invasive ventilation it is unlikely he will survive beyond the next few days. We have therefore moved to palliation. [Wife] is in agreement that it would not be appropriate to intubate and ventilate him. (patient 79)	
1.2 ‘Prof [oncologist] reiterated our conversation we had with [patient] and [husband] this morning about the change from curative to palliative intent. Then we stressed the importance of symptom control, how we were going to involve the palliative care team, and how we would ensure reducing anxiety, distress and any discomfort…’ (patient 63)	
1.3 ‘[patient] agrees to intubation [. . .] but does not want CPR or any heroic measures to prolong her life.’ (patient 59)	
1.4 I talked with [oncologist] yesterday who is keen that we pursue every therapeutic option for [patient] at present and I discussed my reluctance to consider intubation for this gentleman because none of our intensive care treatments were improving his respiratory function [. . .] [Palliative care consultant] talked with the family and indeed I talked to [patient] yesterday who was adamant that he didn’t want further escalation. (patient 16)	
*2. Concomitant prognostication and managing uncertainty*	
2.1 ‘Explained to [husband] that [patient] is gravely unwell at present, and that she may not survive this episode. We have explained to [husband] that we will do everything we can in CCU, and will have more of an idea within the next 24 to 48 hours of how her condition will progress. If she deteriorates further, then we will do everything we can to keep her comfortable… (patient 81)’	
2.2 ‘Unable to wean off FiO2 [oxygen via oscillator mode on ventilator] and respiratory demands continue to increase. [patients]’ condition continued to deteriorate and discussions were had between the family, oncology and CCU team.’ (patient 83)	
2.3 Even were his GVHD to resolve imminently he would still be in multi-organ failure[. . .] (patient 47)	
*3. Parent medical and critical care team decision-making*	
3.1 ‘in view of limited cancer prognosis and, in event of requiring emergency sedation, intubation and ventilation that recovery to the point of acceptable quality of life would be unlikely’ (patient 40)	
3.2 ‘After discussion with [oncologist] and the family we decided not for further active management but continued high quality supportive care’ (patient 84)	
*4. Integrative palliative care*	
4.1 ‘we stressed the importance of symptom control, how we were going to involve the palliative care team, and how we would ensure reducing anxiety, distress and any discomfort. (patient 17)	
4.2 ‘His continuing deterioration is indicative of end of life events and we discussed his management with the palliative care team (patient 15).	
*5. Family-centred versus patient-centred care*	
5.1 ‘Consider discontinuation of NIV at an interval after son’s arrival on Monday. In the meantime—if [patient] wishes us to discontinue the NIV earlier, this would be appropriate—with sensitive communication to the family’ (patient 27)	
5.2 ‘They [family] understand that it is highly unlikely that Mrs D would be able to be weaned from invasive ventilation and agree as to the previous set ceiling of care of NIV [non-invasive ventilation]. They understand she has developed multi-organ failure and that our priority of care now would be solely her comfort.’ (patient 30)	
5.3 ‘Family did not want active treatment if no hope of recovery’ (patient 85)	
5.4 ‘Wife does not want us to try to communicate this to him now but would rather she did that herself if he became more alert later on.’ (patient 80)	
5.5 ‘We will leave her treatment as it as at present instead of withdrawal as her son is in transit from USA.’ (patient 30)	

When initiating end-of-life transitions, where patients had capacity, decisions were made collaboratively with families and teams. When supportive care was deemed appropriate, rather than focusing on active treatment, palliative care input was quickly sought. The excerpt (1.1) exemplifies consensus building as well as highlighting the consensus between teams in the next theme.

*Parent medical and critical care team decision*-*making* referred to how critical care teams sought early joint decision-making with parent medical teams when there was doubt over the possibility of critical illness recovery (theme 3). This planning meant that some patients were transferred out of the unit and received EOLC on the ward.

Poor critical illness related outcomes and cancer prognosis made it straightforward to reach consensus and rationalise decisions. Prognosis was communicated where possible (2.1) and where there was uncertainty regarding outcome from the critical illness perspective, it was more likely that patients died with full intensive care treatments in situ (2.2, 2.3).

Furthermore, teams negotiated to try critical care treatment for a period of time, allowing opportunity for anti-cancer treatments to work, and allowing for a trial of ICU to reverse acute problems. If families were reluctant for EOLC transitions and wished for full active treatment to continue, the intensivists would often continue with the caveat that if there was no improvement in a specified time, they would initiate a transition to end-of-life (2.1).

If families (and patients, when able) were receptive to EOLC transition, then the critical care team acted swiftly to focus on EOLC, either placing a ceiling of care (limitation of treatment) (5.2), or withdrawing treatment (5.1, 5.3).

*Integrative palliative care* referred to how palliative care input was sought on the majority of patients who actually died in critical care, despite the short time to death from withdrawal or limitation of treatment (theme 4). This facilitated the focus on comfort measures, and away from high-intensity treatment at end-of-life, but depended on timely referral. Some intensivists advocated an integrative approach where palliative care became involved early, to assist in advanced care planning.

## Discussion

In this study we found that APACHE II scores, age and levels organ support were found to be associated with patients who were likely to transition from full ICU active care to EOLC. These parameters have also been found to correlate with poor outcomes in other studies [2, 8, 33]. Cancer stage or diagnosis was not associated with transition to EOLC in keeping with findings from other studies [2, 9]. On multivariate analysis, APACHE II score, the need for intubation and non-invasive ventilation were significantly associated with transition to EOLC.

Our results indicate that teams and relatives communicated frequently during the period of transition to EOLC. These findings were supported by the qualitative analysis, which illustrated that families understood the appropriateness of transitions to EOLC, and were keen to avoid suffering when reversal of the underlying condition was not possible. Despite some qualitative documented cases of good communication with families, patients were often too unwell to engage in conversations around transition to EOLC and/or DNACPR once in ICU. Even in this group of patients with known cancer, few patients in the EOLC cohort had been asked and had documented evidence of a preferred place of death. Despite the recent initiatives and published reports that patients want to be involved in decision-making, use of advanced care planning and discussions about EOLC with patients and their families remains infrequent [34–36]. Research indicates that most people want to die at home [37, 38]. However, the majority of our patients in the EOLC cohort died in hospital, with over three-quarters of patients in the EOLC cohort dying in the ICU. For patients with advanced disease it is important that discussions and documentation about ceilings of treatment, prognosis and patient wishes, are done early in the disease course when patients are still well enough to engage, rather than in the ICU. Even in our institution, where the palliative care team forms an integral part of the service this issue remains challenging. In this study the number of patients in the EOLC cohort that we able to participate in discussions about their cancer prognosis (43.2%), DNACPR decision (40.5%) and preferred place of death (10.5%) during their ICU stay was low. Whilst this may just represent of the number of patients who presented with acute, unforeseen deterioration in their clinical condition unrelated to their cancer progression, it highlights the need for early palliative care involvement and advanced EOLC planning. There may be frequent opportunities for discussion during outpatient visits and in-patient admissions. A recent study indicated that patients having palliative chemotherapy towards the end-of-life are more likely to die in ICU [39]. When the focus of care is on survival, health professionals are often reluctant to discuss prognosis or plans in the event of deterioration as it may be considered unnecessarily distressing to patients [40]. Yet, for some patients, earlier discussion of prognosis, treatment options and EOLC wishes may mean they are better informed to make decisions about their care. Also, Rees et al. [41] did not find that these discussions caused distress for most of their patients.

Often the decision to make a patient DNACPR or to transition to EOLC was made within 48 h of ICU admission, following a ‘trial of ICU’. Tools have been developed for use in patients admitted to ICU to improve communication and palliative care [42]. These tools require further validation and review but may be used to support medical decision-making and prompt early discussions between clinicians, patients and their families about patients’ wishes, potential therapeutic options and anticipated outcomes.

Few patients (10%) who transitioned to EOLC during their ICU stay were already known to the palliative care team prior to ICU admission. This may be due to the fact that many patients were treated with curative intent, and had not required palliative care intervention prior to their critical illness. Our qualitative data suggests that intensivists had the skills to manage symptoms and support families. Even when the patient was admitted to the ICU, the palliative care team were only involved in 53% of patients in the EOLC cohort. Early palliative care involvement has been associated with a lower proportion of deaths occurring in the ICU [43].

Based on clinical documentation, spiritual, psychological and welfare support was offered to patients/families at end-of-life in only a third of cases. It may have been that support was offered and declined, that the ICU team was already giving adequate support to patients and their families, or that these needs were not consistently considered. Family satisfaction with EOLC decision-making has been closely associated with documented discussion of patient wishes and spiritual needs [44]. In the United Kingdom, one of the useful aspects of the Liverpool Care Pathway (LCP) was that it acted as a prompt for staff to consider emotional, spiritual and psychological support at the end-of-life [27, 45]. In the post-Liverpool Care Pathway era, we need to ensure these vital quality indicators of good EOLC are considered, offered if appropriate and clearly documented. There are local and national initiatives underway to develop such tools [46].

One of the limitations of this study was that the data were collected retrospectively therefore the results not only reflect the processes of care, but also were reliant on the quality of documentation. For example, only specific aspects of the documented content of communication were evaluated and the quality of communication could not always be ascertained from the documented data. Also, the binary assessment of some of the quality markers may have been, in some circumstances, insufficient to assess quality of the care process provided but in some cases could be further explored in the qualitative analysis. It could be also argued that the findings are limited to the results of a single, tertiary centre; however, the authors believe that the findings may have wider applicability, as the themes identified are common to findings from other studies which have included general ICUs [14, 15]. The small sample size and possible existence of unmeasured confounding factors may have impacted on some of the conclusions drawn. Finally, only a limited assessment of end-of-life symptoms, and subsequent management, was undertaken; given the retrospective nature of the study. The use of defined assessment or outcome scales may have provided more robust information about the quality symptom management.

## Conclusions

Larger studies are required to identify and validate clinical factors that may help identify patients with cancer who are likely to transition to EOLC during their ICU stay. Although qualitative and quantitative analysis of the records demonstrated that there was communication and collaborative decision-making between medical teams, patients and families during transition to EOLC, 40% of patients were too unwell to engage in conversations about preferences for care. This highlights the need for early advanced care planning, ideally prior to ICU admission. Our findings, although arguably those from a single centre, do have widely applicability, and demonstrate that there is a clear need for the development and validation of tools to support high-quality patient-centred EOLC in a critical care setting, across all domains.
